# Border malaria: defining the problem to address the challenge of malaria elimination

**DOI:** 10.1186/s12936-023-04675-3

**Published:** 2023-08-21

**Authors:** Xiaohong Li, Robert W. Snow, Kim Lindblade, Abdisalan M. Noor, Richard Steketee, Regina Rabinovich, Deyer Gopinath, Elkhan Gasimov, Pedro L. Alonso

**Affiliations:** 1 Global Malaria Programme, World Health Organization, Geneva, Switzerland; 2 Kenya Medical Research Institute (KEMRI)-Wellcome Trust Research Programme, Nairobi, Kenya; 3 Centre for Tropical Medicine & Global Health, Nuffield Department of Clinical Medicine, University of Oxford, Oxford, UK; 4 PATH, Seattle, USA; 5 Barcelona Institute of Global Health, Barcelona, Spain; 6 World Health Organization Country Office, Bangkok, Thailand

**Keywords:** Border malaria, Cross border, Malaria importation, Malaria elimination

## Abstract

Border malaria is frequently cited as an obstacle to malaria elimination and sometimes used as a justification for the failure of elimination. Numerous border or cross-border meetings and elimination initiatives have been convened to address this bottleneck to elimination. In this Perspective, border malaria is defined as malaria transmission, or the potential for transmission, across or along shared land borders between countries where at least one of them has ongoing malaria transmission. Border malaria is distinct from malaria importation, which can occur anywhere and in any country. The authors’ analysis shows that the remaining transmission foci of malaria-eliminating countries tend to occur in the vicinity of international land borders that they share with neighbouring endemic countries. The reasons why international land borders often represent the last mile in malaria elimination are complex. The authors argue that the often higher intrinsic transmission potential, the neglect of investment and development, the constant risk of malaria importation due to cross-border movement, the challenges of implementing interventions in complex environments and uncoordinated action in a cross-border shared transmission focus all contribute to the difficulties of malaria elimination in border areas. Border malaria reflects the limitations of the current tools and interventions for malaria elimination and implies the need for social cohesion, basic health services, community economic conditions, and policy dialogue and coordination to achieve the expected impact of malaria interventions. Given the uniqueness of each border and the complex and multifaceted nature of border malaria, a situation analysis to define and characterize the determinants of transmission is essential to inform a problem-solving mindset and develop appropriate strategies to eliminate malaria in these areas.

## Background

Globally significant progress has been made towards malaria elimination. The number of countries reporting fewer than 10,000 malaria cases per year—an arbitrary indicator that a country is on the verge of elimination—has increased from 26 to 46 over the past two decades [1]. In the same period, the number of countries reporting fewer than 100 indigenous cases per year has increased from 6 to 26. One of the most frequently cited challenges of malaria elimination is “border malaria” or “cross-border malaria” [2]. Discussions on the challenges of international borders in malaria elimination started in the global malaria eradication programme (GMEP) [3] and continued in numerous border or cross-border meetings over decades as countries and regions pursue their elimination goals. The World Health Organization (WHO) South-East Asia Region declared its regional elimination goal in 2017, but the persistent malaria transmission in border areas has become a critical concern [4]. The significance of border malaria is reflected in the increasing number of regional elimination initiatives within which cross-border coordination is often a component [5]. A recent study considered that malaria elimination took longer in territories with land boundaries, after evaluating successful malaria elimination trajectories of 42 malaria programmes [6].

To determine whether the remaining malaria transmission is spatially distributed in border areas when countries became close to elimination, data from malaria-eliminating countries were collected and analysed. Countries that are included in the two global elimination initiatives, the Elimination-2020 initiative and the Elimination-2025 initiative [7], and those that have been certified malaria-free by the WHO are included in the analysis. The studied time points for E-2020 and E-2025 countries were the time when they joined the initiatives, as they were considered to have the potential to achieve elimination by 2020 or 2025 at that time. For certified countries, the studied time point was 12 or fewer than 12 years prior to the detection of the last indigenous cases, using Cohen’s analysis on the threshold of the last mile of malaria elimination [6]. Data were normally retrieved from the World Malaria Report database, if available, or extracted from literature or grey literature.

To define the characteristics of border malaria and factors affecting malaria transmission in border areas, a search was conducted on publicly available databases including PubMed and Google Scholar with the following keywords: Cross-border malaria; Border malaria; Border transmission; Migrants; malaria importation. The time covered the period between 2007 and 2023. The WHO publications and meeting reports in IRIS (IRIS Home, who.int) and the WHO archives were searched using the keywords “border” OR “cross-border” plus “malaria”. The official websites of the United Nations Development Programme, the United Nation Refugee Agency and national statistics were consulted for information on the economy, ecology, development, demography and political context in the concerned border areas of the malaria eliminating countries and their neighbours. The review included those literature and grey literature describing malaria ecology, epidemiology, determinants and contextual factors for malaria transmission such as transmission potential, health systems, social and economic development, demography and challenges in implementing malaria interventions in the concerned border areas of eliminating countries, which are defined as countries that have declared a national malaria elimination goal and report fewer than 10,000 cases annually. Publications describing malaria importation or issues related to mobile and migrant populations that do not affect the vicinity of international land borders were excluded. The data and information extracted were verified through interviews with key informants, including national malaria programme managers, senior researchers, and former and current World Health Organization staff who had visited the countries or areas in question. Their role was to confirm and verify malaria transmission in border areas during the study period and contextual factors such as the political context in border areas.

Given that the terms “border malaria” and “cross-border malaria” have been used inconsistently, the definition established by a WHO Evidence Review Group is used with slight modification. Here border malaria is defined as “malaria transmission or potential for transmission that takes place across or along land borders between countries sharing a border in which at least one of them, has ongoing malaria transmission” [8]. While this definition purposely avoids providing a precise distance as operational differences are expected in affected countries, it delimits the geographical scope of border malaria to areas in the vicinity of international land borders. The definition excludes malaria importation to interior areas far away from the international land border, through sea borders or airports or into island countries. For example, Sri Lanka does not have border malaria as the country is an island, although significant importation can happen from neighbouring India. Similarly, malaria importation by Mozambican mine workers into the province of Gauteng in South Africa is not characterized as border malaria, because Gauteng is hundreds of kilometres away from the international land border. Both two situations are malaria importation. The management of malaria importation might be challenging, but it is the sole responsibility of the concerned Government. Border malaria is differentiated from malaria importation and the two entail different policies.

In this Perspective, the authors first seek to answer the question whether the last few malaria transmission foci of malaria-eliminating countries tend to occur in border areas. Secondly, important features of border malaria were discussed and possible explanations why “border” represented the last mile of malaria elimination were provided. Finally, the authors proposed recommendations and suggested action items that may help affected countries to tackle border malaria and move the border malaria agenda forward.

## Is border malaria a real problem?

**Fig. 1:**
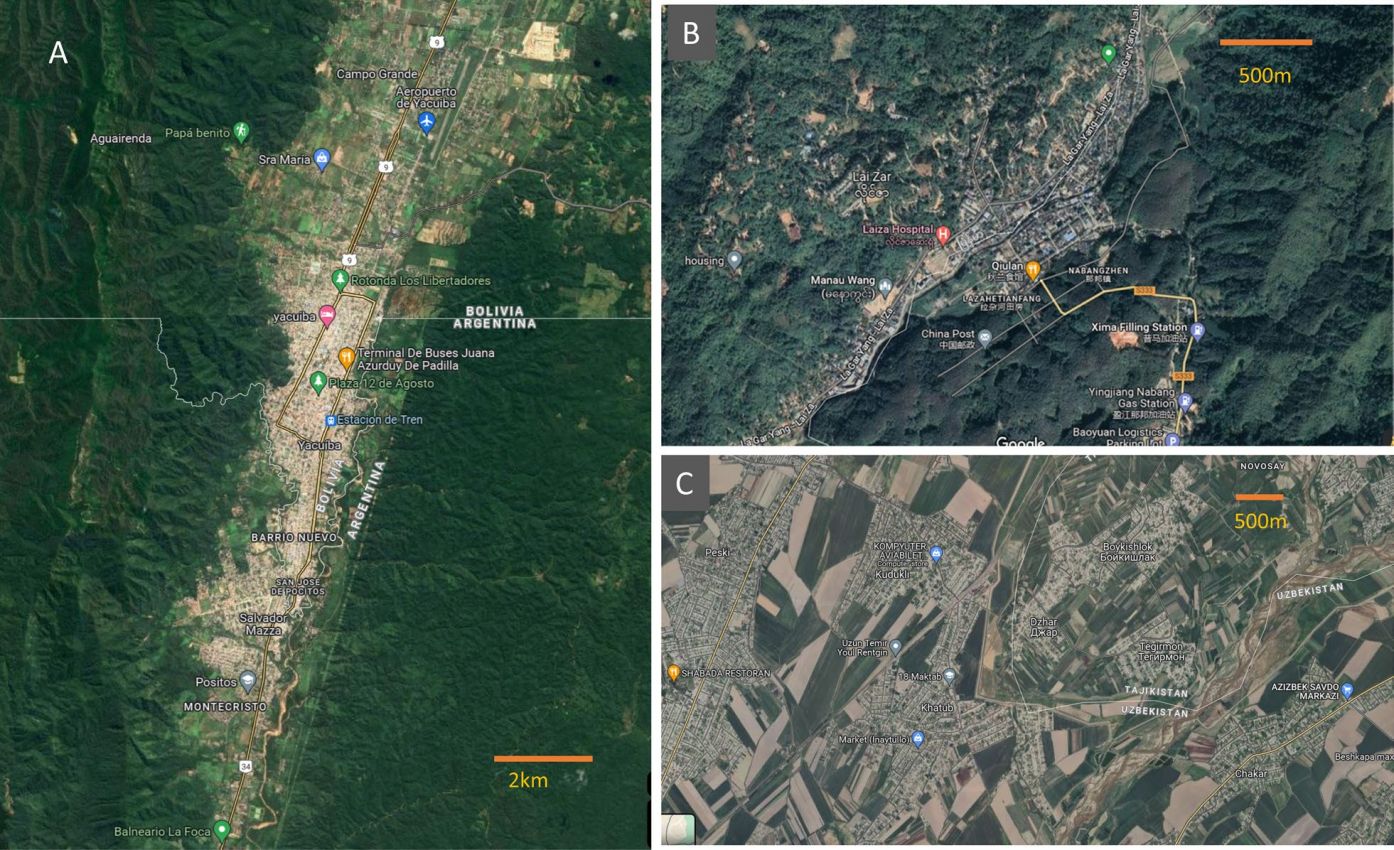
Bird view of malaria foci that were shared by two neighbouring countries. **A** Yacuiba in Bolivia and Salvador Mazza in Argentina; **B** Laiza in Myanmar and Nabangzhen in Yingjiang County, Yunnan, China; **C** villages of Uzun district in Uzbekistan and Tursunzoda district in Tajikistan)

“Mosquitoes do not carry a passport to cross the border” is frequently cited by officials and programmes to justify the status quo or failure to eliminate malaria transmission within national borders. Wherever settlements are very close to international borders (closer than a few hundred metres) and effectively form a shared focus (Fig. 1), anopheline mosquitoes that carry the malaria parasite can fly across the border and cause human infections [9]. However, anophelines normally are unlikely to fly more than 1 km under natural conditions [10, 11]. They are energy-efficient and seek blood meals from sources that are nearby. Therefore, although anophelines flying over the border are sometimes part of the border malaria problem, this is not a common and major issue. The epidemiological factors that support malaria transmission in border areas are not different from those that apply elsewhere: the presence of vectors, human hosts and parasites. As such is border malaria a unique epidemiological construct?

**Fig. 2:**
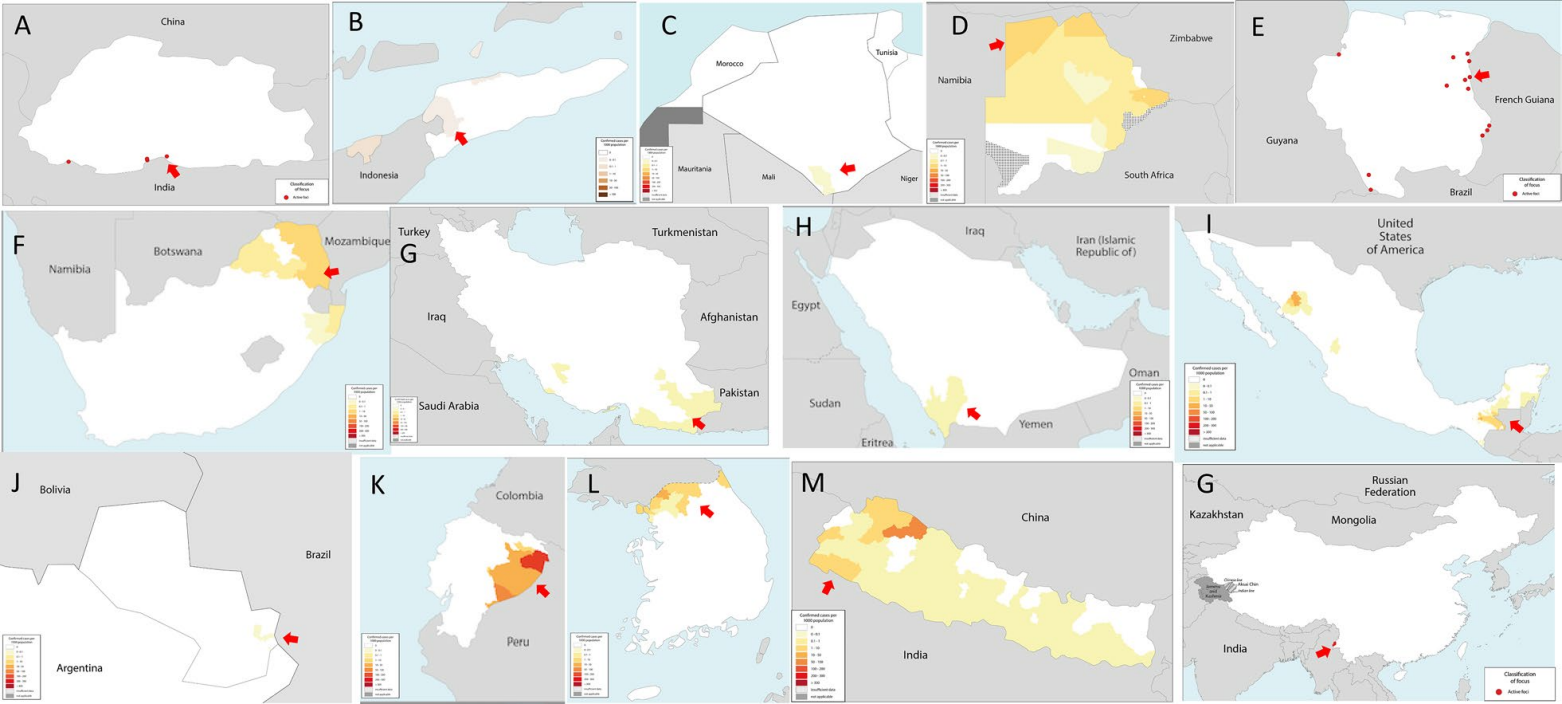
Malaria transmission in elimination-2020 countries. Maps show E-2020 countries that had malaria transmission in border areas, pointed by red arrows. **A** Bhutan (2017); **B**. Timor Leste (2016); **C** Algeria (2012); **D** Botswana (2018); **E** Suriname (2018); **F** South Africa (2018); **G** Iran (2017); **H** Saudi Arabia (2018); **I** Mexico (2018); **J** Paraguay (2010); **K** Ecuador (2018); **L** South Korea (2018); **M** Nepal (2018); **G** China (2016). Red dots in **A**, **E** and **G** show active foci while **C** and **J** coloured the areas with indigenous cases. The rest maps show areas with reported malaria cases, local transmission pointed by red arrows and confirmed by national malaria programmes. Maps are not to the scale. (Source of data: World Malaria Report)

In 2016, the WHO identified 21 countries—referred to as E-2020 countries—that had the potential to eliminate malaria by 2020, based on the number of cases, the declared malaria objectives of countries, and informed opinions in the field [6]. Of these E-2020 countries, 19 share international land borders with endemic countries, and 14 have identified their last few indigenous cases in areas in the vicinity of international land borders (Fig. 2). The E-2020 initiative has recently transitioned to E-2025 and most of them also observed their last few transmission foci near international land borders (Table 1). The fact that international land borders represent the last mile of malaria elimination is not recent. A retrospective review conducted by the authors of countries that have been certified as malaria-free by the WHO since the 1950s revealed a similar picture (Table 1). Out of 22 countries, 15 had the last few transmission foci in the border areas. The challenge posed by international land borders to disease elimination is not unique to malaria, discussed extensively in eradication of smallpox, polio, guinea worm disease and the elimination of other neglected tropical diseases [12–15]. It is neither limited to countries that only had very low malaria transmission [16]. However, the high prevalence of the border problem in the last stage of malaria elimination and its persistence over time remain striking and intriguing.

**Table 1:** E-2025 countries and certified malaria-free countries that had border malaria [17–26]

Country characteristics		E-2025 countries	Countries certified malaria-free by WHO
Countries sharing international land borders with other countries	Last few foci in border area	Belize, Bhutan, Botswana, Democratic People’s Republic of Korea, Ecuador, Honduras, Iran (Islamic Republic of), Mexico, Nepal, Panama, Republic of Korea, Suriname, Thailand, Timor-Leste, Saudi Arabia, South Africa, Guatemala, Costa Rica, French Guyana	Argentina, Armenia, Bulgaria, China, Kyrgyzstan, Paraguay, Poland, Romania, Spain, Portugal, Turkmenistan, United Arabic Emirates, Uzbekistan, Azerbaijan, Tajikistan
	Last few foci not in border area	Dominican Republic, Eswatini, Malaysia	El Salvador, Hungary, Italy, Morocco, Netherlands, United States of America, Former Yugoslavia
Island countries		Cabo Verde, Comoros, Sao Tome and Principe, Vanuatu	Cyprus, Australia, Cuba, Dominica, Grenada, Jamaica, La Réunion (region of France), Maldives, Mauritius, Saint Lucia, Singapore, Sri Lanka, Trinidad and Tobago

## Why does “border” often represent the last mile of malaria elimination?

### Receptivity or transmission potential in border areas

Malaria is an ancient disease that afflicted human’s early ancestors, if not hominids. As a major human killer, malaria has had a profound influence on the history and the pattern of urbanization [18, 27]. The process of civilization differentiated “central” or “interior” areas, where settlements and civilization were established earlier, from “remote” and “periphery” areas, where natural conditions were unfavourable for human habitation. A study of China over a period of 2000 years revealed that the climatic potential for the transmission of *Plasmodium falciparum*, the deadliest species of the malaria parasite, has influenced the spatial distribution of urbanization [27]. The effect is still visible today: areas with a high potential for malaria transmission are relatively insignificant in terms of their urban population and economic activity, even years after the elimination of *P. falciparum* [27].

**Fig. 3:**
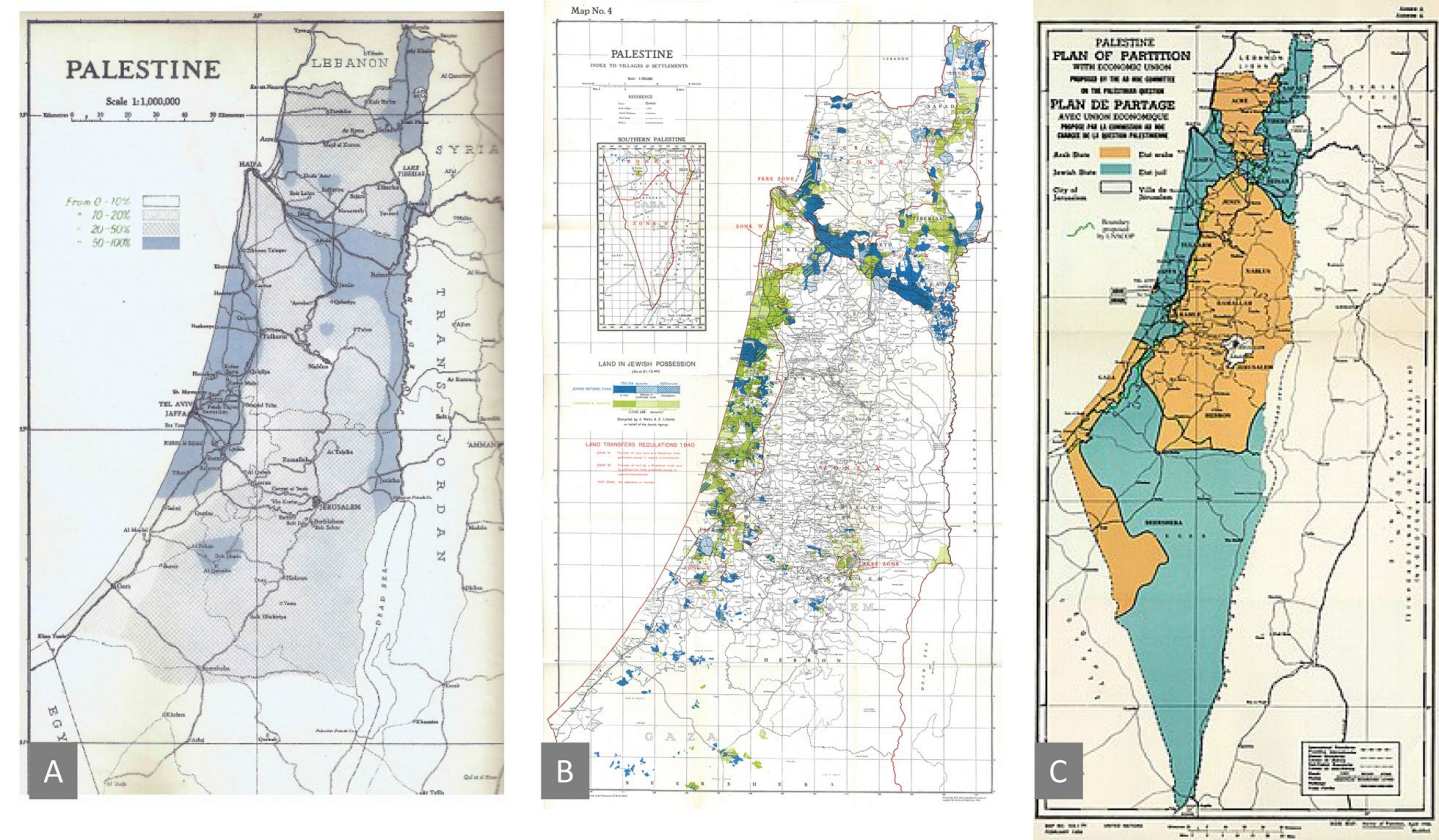
Historical maps of Palestine. **A** Spleen enlargement rates indicating severe malaria areas in Palestine in 1920 (Source: Palestine Departmen2 t of Health 1941); **B** Jewish settlement areas in Palestine (coloured in blue and green) (source,UN 1947); **C** 1947 UN Partition Plan for Palestine (Source: UN, 1947)

Malaria may play a role in defining the boundaries between states. The political boundaries between the Arab and Jewish States, which were delineated in the United Nations Partition Plan for Palestine in 1947, coincided with the boundaries of severity of malaria in Palestine in 1920 (Fig. 3). At that time, Arabs had already settled in the low malaria transmission areas of Palestine, leaving the high malaria endemic areas sparsely populated. After the arrival of Jews, significant efforts were made to eliminate malaria, which resulted in a more habitable land and the birth of state of Israel [28]. Here “border” is a footprint of the interplay between malaria, man and his environment.

Many border areas in the Greater Mekong Subregion and in the American region are forest areas with an ecological environment favouring malaria transmission. Current vector control tools are unable to fully control these vectors, contributing to the general higher receptivity (i.e. level of underlying suitability for transmission) in forest areas. The districts bordering Yemen in Saudi Arabia fall in the Afrotropical zone, harbouring an efficient vector, *Anopheles arabiensis*. This could explain, at least partly, why border areas in Saudi Arabia still had malaria transmission years after the rest of the country has eliminated the disease [29] (Table 2). The northern border districts in Namibia have been a focus of stable, high malaria transmission even before the border with Angola was demarcated [30]. Because receptivity is by far the greatest factor determining the magnitude of the challenge in reaching elimination [31], it is not surprising that border areas with high intrinsic transmission potential are the last to eliminate malaria.

**Table 2:** Characteristics of border malaria

Factors		Examples	Frequency	Refs.
**Receptivity in border areas (intrinsic transmission potential)**				
High original endemicity		Iran border, China (Yunnan) border, Saudi Arabian border, Namibian border	+ +	[29, 30, 32, 33]
More efficient vector(s)		Saudi Arabia, China (Yunnan), Iran	+ +	
Being forest areas		Greater Mekong Subregion countries, American countries	+ +	
**Social, economic and political environment**				
Border areas being poorer areas in the eliminating country		Namibia, Saudi Arabia, China (Yunnan), Iran (Baluchistan),	+ + +	[29, 33–35]
Conflicts, political unrest, security issues		Saudi Arabia–Yemen border, Bhutan–India border, China–Myanmar border, Bangladesh–Myanmar border, Thailand–Myanmar border, Pakistan–Iran border	+ +	
**Malaria epidemiology**				
Straddled foci across international land borders		The borders between Argentina and Bolivia, Peru-Ecuador, China-Myanmar, Bhutan-India, Tajikistan-Afghanistan	+ +	
Two sides of the border are within one ecological zone		Greater Mekong Subregion countries, American countries	+ + +	
Existence of a transmission gradient in two neighboring countries		All above mentioned borders	+ + +	
**Cross-border movement**				
Short-term, cyclical, frequent		China–Myanmar border, Bhutan–India border, Iran–Pakistan border, Namibia–Angola; Saudi Arabia–Yemen	+ + +	
Borders are not controlled		As above		

### Social and economic development, and political unrest

Not all border areas are more receptive to malaria transmission than interior areas as the demarcation of international borders is complex (Table 1). However, border areas are often the poorer, if not the poorest, areas in a country (Table 2). For example, border areas in Sistan and Baluchestan province in Iran, Chiapas state in Mexico, and Kawango in Namibia, were all the poorest regions in their countries with persistent malaria transmission [32, 34, 35]. Yunnan province had the 2nd lowest GDP per capita in China (data in 2017) and the income levels were significantly lower along the border areas than in the interior counties within the Province [36]. The widespread poverty in border regions could be due to the negative impact of malaria on urbanization and economic as described above. In addition, border areas happened to be the battlefields in history, remote areas with difficult terrain, and home of the marginalized populations such as indigenous and ethnic groups or nomadic. Investment in border areas tends to delay or does not exist as this might entail political, social and environmental risks and uncertainty. Indeed, some borders were only demarcated and settled years after the countries declared independence. A case in point is the border between Saudi Arabia and Yemen, which was only settled in 2000 while the Kingdom of Saudi Arabia was established in the 1930s [29]. Many borders are still under dispute today. Political unrest and security issues are frequently found in border areas [37]. Poverty and political unrest negatively impacted infrastructure and health systems and, therefore, hinders efforts to eliminate diseases of many kinds. However, because economic development is found to be the major driver of declining entomological receptivity, a key determinant for malaria elimination [31], the impact of the low economic status is expectedly more prominent and profound on malaria.

### Cross-border movement and challenges in implementing malaria programmes

Although most international borders are regulated and require procedures for entry, cross-border movements may be frequent, especially where the borders are porous, and the control of movement is not feasible. Movements are often short-term and cyclical, rather than involving longstanding changes in residence. People cross the border frequently to visit families or for schooling because they often share a common lineage and social structure with communities across the border [29, 33]. Movement can also occur for reasons relating to business, seeking health care or security triggered by disparities between neighbouring countries in social and economic development, access to and quality of health services or the difference in laws and regulations.

The contiguous regions often have different levels of endemicity, which forms a transmission gradient across border. For example, the annual parasite incidence (API) in the border regions in Myanmar was 60 times of that in Yunnan border counties in 2008 [38]. Tumbes Region of Peru borders El Oro in Ecuador and the average API from 1990 to 2012 in Tumbes was more than 3 times of that in El Oro [39] (Table 2). Through cross-border movement, malaria parasites are imported from the area with a higher transmission to its neighbour where elimination is closer (or vice versa) which demands rapid detection and response to prevent onward transmission by imported cases. However, although a common concern, cross-border movements are often difficult to quantify and the level of importation resulting from such movements is unknown. Accordingly, it is hard to measure the degree to which cross-border movement has fueled malaria transmission, which is determined not only by the level of importation but also by entomological receptivity and the response of the health system in the recipient areas. Nevertheless, short-term, frequent, and cyclical cross-border movement, unlike the unidirectional and less frequent migration in interior areas, presents unique challenges for the effective implementation of interventions, including surveillance and case classification. The contiguous areas share a common ecology, with frequent mixing of people, parasites, and vectors. From an epidemiological standpoint, the political border does not cut off the continuation of the transmission focus or transmission zone (Fig. 1), but it effectively demarcates the territories and the sovereignty of two political entities, each having their own health systems, health priorities and development agendas. Hence, the implementation of interventions is often unequal and not coordinated across international borders which could compromise the impact in some circumstances.

Cross-border movement is not the only challenge for malaria elimination in border areas. The population at risk of malaria in border areas could be communities that have different health-seeking behaviour or limited access to health care, such as indigenous population, forest goers, gold miners, legal or illegal migrant workers or military personnel. The health system is often weaker in border areas—consistent with the low development status and remoteness of border areas as described above. Inadequate access to health care could be due to the limited infrastructure in the remote border areas; lack of trained health staff; the existence of physical, financial, or other barriers for people to seek health care; and low utilization of malaria interventions. These same factors impact the quality of surveillance and other malaria interventions, which are also critical for malaria elimination. Only after the populations at risk of malaria and the factors that affect their access to and utilization of malaria services are identified can strategies and interventions be found to address these issues.

## Conclusion and recommendations

Border malaria is not a unique epidemiological construct. Interventions that are effective to reduce and eventually eliminate malaria elsewhere remain valid in border areas. Without universal access to health services, robust surveillance and response, and effective implementation of an elimination programme, malaria elimination will not be achieved anywhere including in border areas. More efficient tools as well as economic development are needed to accelerate the decline in malaria receptivity and shorten the time it will take to reach elimination. The challenges for malaria elimination in border areas contribute to the long tail of elimination. The authors propose the following recommendations to help affected countries to tackle border malaria and action items (Table 3) to move the border malaria agenda forward.

**Table 3:** Action items for border malaria

Early planning and management	To ensure comparable progress towards the sustainable development goals in border areas,
	• Direct additional resources to border areas to improve the infrastructures, the access to health services and the strength of health system
	• Establish and implement policies that provide inclusive malaria services for all populations, including non-citizens, undocumented migrants, and refugees
	• Develop high-level political strategies, engage stakeholders and take prompt actions to return peace, justice, and access to healthcare in border areas with conflicts and political instability
	• Address major social determinants of health such as education, wealth, employment, and social protection
Define the malaria problem in border area	Perform a situation analysis to define and characterize the malaria problems in border areas which could include
	• Delineate the geographical boundaries of malaria transmission through the review of available data and field visits; identify the source of malaria importation and their destination; and differentiate border malaria from malaria importation
	• Review information on determinants and contextual factors for malaria transmission at borders including malaria ecology, geography, environmental features, demographic characteristics of interested populations, cross border movements and political context
	• Assess the availabilities and access to health care, the coverage and quality of surveillance, the implementation of malaria interventions and their impact
	• Assess whether a shared transmission focus was formed across borders
	• Determine the reasons underpinning the inadequate access to health care, inadequate surveillance and suboptimal implementation of malaria intervention wherever relevant, determine whether the cross border collaboration is appropriate, and develop strategies and interventions
Cross-sector and cross-border collaboration	• Cross-sector collaboration
	– Establish mechanisms for intra- and intersectoral information-sharing
	– Coordinate and synergize the efforts for malaria control, elimination and prevention [40]
	• Cross-border collaboration
	– Jointly conduct a situation analysis with neighbours to identify areas of interest for cross-border collaboration. This can help better define the geographical boundaries of malaria transmission and determine the source and destination of importation where relevant
	– Engage key stakeholders, including a neutral third party, to establish the information-sharing mechanism and to identify joint activities (e.g. synchronize vector control) to maximize the impact of interventions and achieve the malaria goals
	– Reach a consensus on cross-border collaboration
	– Mobilize resources for implementation. Jointly monitoring and evaluation to assess impact

### Situation analysis to define the exact problem

Border malaria is a complex and multifaceted issue. Every border is unique in its malaria epidemiology, social, economic, and political determinants. It is, therefore, crucial to define and characterize the border malaria problem clearly. An essential starting point is a situation analysis which must include defining the geographical boundaries of where transmission is taking place, identifying the infected population, and determining the source and destination of importation. Not all importation is relevant to an analysis of border malaria. Some countries have substantial malaria importation, including into areas with transmission potential, without constituting a border malaria problem as the destination of imported cases is far away from the administrative border.

The analysis should also include: (i) a review of data on original endemicity, available information on geography, natural and environmental features of the border and border areas,; (ii) malaria ecology, including parasite and vector species, vector bionomics, temperature and rainfall, incidence and prevalence, and risk groups; (iii) an assessment of access to health services which can be adapted to the existing tool [41]; (iv) malaria interventions, such as case management, vector control and surveillance; their implementation, coverage and quality [42]; (v) demographic characteristics of the populations of interest and cross-border movement including the motives for and patterns of movement; (vii) cross-border collaboration and cross-sector collaboration, if any; (viii) the political situation.

### Cross-sector and cross-border collaboration

The term “border malaria” implies that cross-border collaboration and coordination are needed to solve the malaria problem at borders. International collaboration is unlikely to be a single process, but should focus on the specifics of and the solutions to the identified challenges. Sharing information will be critical to help the malaria programme plan and prepare the response, but cross-border coordination will likely take different forms to be effective across different borders, depending on the existence of a shared transmission focus, the relatedness of the populations on both sides of the border, the level of transmission, the impact that cross-border migrants might have on transmission and the political context among others. It is equally important that within a country, the collaboration between the health sector and other key sectors (e.g., border security, economic and civil society, education and others) is fostered to synergize efforts and to achieve the effectiveness and efficiency of malaria interventions when dealing with both malaria importation and border malaria.

### Early planning and management

Given that the border area may be the last place where transmission will be interrupted, early planning and the development of health system will be required to shorten the long tail of malaria elimination. Necessary additional resources should be directed to border areas to improve infrastructure and the access to health service, improve human uptake of prevention services, strengthen health system, and assure timely and accurate information to track progress and respond when and where necessary. Early attention and investment are needed to ensure that border areas are not neglected in terms of economic growth, education, improvement of living and working conditions and accelerating progress towards the Sustainable Development Goals. With a well characterized situation analysis in place, early planning should engage donors and partners who are involved in the essential health services delivery in fragile, conflict-affected border areas to promote sustainable service delivery in the long term.

The lingering transmission in border areas reflects the limits of the current tools and interventions in controlling and eliminating malaria as well as the challenges of implementing them in a complex environment. “Border areas” often represent the last mile of malaria elimination and can be hard places for a variety of reasons. The application of anti-malarial measures can only achieve the expected results in controlling and ultimately eliminating malaria when the planning and resources address the necessary social cohesion, general education, basic health services, community economic conditions, and information sharing among partners at the border. The speed of malaria elimination will depend on early attention in the hardest places—especially border areas.

## Data Availability

Comment using data from published literature and grey literature including those in World Malaria Report (https://apps.who.int/iris/).
